# Noninvasive Prenatal Paternity Testing with a Combination of Well-Established SNP and STR Markers Using Massively Parallel Sequencing

**DOI:** 10.3390/genes12030454

**Published:** 2021-03-22

**Authors:** Xuefeng Shen, Ran Li, Haixia Li, Yu Gao, Hui Chen, Ning Qu, Dan Peng, Riga Wu, Hongyu Sun

**Affiliations:** 1 Faculty of Forensic Medicine, Zhongshan School of Medicine, Sun Yat-Sen University, Guangzhou 510080, China; shenxf3@mail2.sysu.edu.cn (X.S.); liran6@mail2.sysu.edu.cn (R.L.); lhaix@mail3.sysu.edu.cn (H.L.); chenh258@mail2.sysu.edu.cn (H.C.); quning@mail2.sysu.edu.cn (N.Q.); pengdan5@mail2.sysu.edu.cn (D.P.); 2 Guangdong Province Translational Forensic Medicine Engineering Technology Research Center, Sun Yat-Sen University, Guangzhou 510080, China; 3 Department of Obstetrics, The Sixth Affiliated Hospital of Sun Yat-Sen University, Guangzhou 510630, China; gaoy57@mail.sysu.edu.cn

**Keywords:** forensic genetics, noninvasive prenatal paternity testing, short tandem repeat, single nucleotide polymorphism

## Abstract

Cell-free fetal DNA (cffDNA) from maternal plasma has made it possible to develop noninvasive prenatal paternity testing (NIPPT). However, most studies have focused on customized single nucleotide polymorphism (SNP) typing systems and few have used conventional short tandem repeat (STR) markers. Based on massively parallel sequencing (MPS), this study used a widely-accepted forensic multiplex assay system to evaluate the effect of noninvasive prenatal paternity testing with a combination of well-established SNP and STR markers. Using a ForenSeq DNA Signature Prep Kit, NIPPT was performed in 17 real parentage cases with monovular unborn fetuses at 7 to 24 gestational weeks. Different analytical strategies for the identification of paternally inherited allele (PIA) were developed to deal with SNPs and STRs. Combined paternity index (CPI) for 17 real trios as well as 272 unrelated trios was calculated. With the combination of SNPs and A-STRs, 82.35% (14/17), 88.24% (15/17), 94.12% (16/17), and 94.12% (16/17) of real trios could be accurately determined when the likelihood ratio (LR) threshold for paternity inclusion was set to 10,000, 1000, 100, and 10, respectively. This reveals that simultaneous surveys of SNP and STR markers included in the ForenSeq DNA Signature Prep Kit offer a promising method for NIPPT using MPS technology.

## 1. Introduction

Prenatal paternity testing is of great importance in certain situations, such as in the investigation of pregnancy due to rape or incest. One possible method is to perform paternity testing through amniocentesis and chorionic villus sampling at the gestational age of week ten and beyond [1,2,3]. However, the use of utensils through the cervix or abdomen is invasive, and such sampling can lead to a risk of complications, including but not limited to limb reduction defects [4], fetal respiratory disturbances [5], and miscarriage [6].

Fortunately, the discovery of cell-free fetal DNA (cffDNA) in maternal plasma [7] enables the possibility of noninvasive prenatal paternity testing (NIPPT) [8]. Aside from the noninvasiveness of cffDNA-based paternity testing that avoids risks associated with invasive procedures, encouraging results also showed that cffDNA can be detected at the gestational age of week four at the earliest [9,10]. Therefore, the development of highly sensitive and accurate NIPPT methods which are easy to popularize is crucial for forensic genetic researchers.

Short tandem repeat (STR) is a conventionally used genetic marker in paternity testing, and capillary electrophoresis (CE)-based STR length polymorphism analysis is the most commonly utilized typing method. Thus, early attempts in NIPPT began with STR; however, investigators were only able to detect few numbers of informative paternally derived alleles from autosomal STRs (A-STRs) [11,12]. On the one hand, stutter artifacts were produced frequently during the STR genotyping, which may influence the identification of paternally derived alleles. Since cffDNA generally comprises <20% of cell-free DNA (cfDNA) in maternal plasma [13], masking of cffDNA by dominant maternal DNA further complicated the testing. On the other hand, cffDNA is usually <160 bp in length [14,15], which is shorter than many STR amplicons detected by the CE method.

In recent years, many genetic markers, such as single nucleotide polymorphism (SNP) [16], DNA methylation [17], deletion/insertion polymorphism-short tandem repeat (DIP-STR) [18] and microhaplotype [19], were used to develop NIPPT methods. Among these, SNP typing by high-throughput sequencing or microarray technology was the most frequently used method in NIPPT due to their short amplicon sizes. For example, Chang et al. established a 5457 SNP typing system based on massively parallel sequencing (MPS) for NIPPT [20]. Qu et al. selected 1795 SNPs and performed NIPPT on the basis of the Illumina HiSeq platform [21]. However, the polymorphism of SNP markers was limited and thousands of markers are recommended to achieve a higher accuracy [16]. Moreover, whether the popular SNP markers or the new microhaplotype markers and self-designed panels are unfavorable for data communication and utilization of public reference database lacks evaluation. As a result, there is a lack of widely acknowledged panels and unified evaluation criteria, resulting in uncertainty on the evidence value of NIPPT.

STR markers are the main markers for exclusion-based relationship analysis and many STR DNA databases have been built and are still going on. If the advancement of well-established STR markers could be taken, the application of NIPPT could be broadened. To achieve this goal, both the problem of long amplicon size and high rate of stutters using the conventional CE-based STR typing method should be solved, so that paternally derived alleles could not only be detected but also be distinguished easier from stutters. Fortunately, MPS enables genotyping of combinations of multiple types (e.g., autosomal STRs, X-STRs, Y-STRs and SNPs) and large numbers of markers with shorter amplicon lengths than the CE method. Among MPS panels developed for forensic research, the ForenSeq DNA Signature Prep Kit (Verogen., San Diego, CA, USA), which is based on Illumina Miseq FGx platform, was widely used and evaluated [22]. Also, a sequence simplification method for MPS-based STR sequence polymorphism analysis was developed, which may bring improvement for mixture deconvolution under stutter interference [23]. For unbalanced mixtures, the minor contributor’s alleles could be identified even when the ratios were as low as 1:1000. Therefore, we investigated the utility of the ForenSeq DNA Signature Prep Kit in NIPPT and the usefulness of this sequence simplification strategy, to test the effect of noninvasive prenatal paternity testing with a combination of well-established SNP and STR markers.

## 2. Materials and Methods

### 2.1. Sample Collection and DNA Extraction

A total of 17 pregnant women with singleton pregnancies, including eight in their first trimester (7 to 12 gestational weeks) and nine in their second trimester (13 to 24 gestational weeks), were enlisted at the Sixth Affiliated Hospital of Sun Yat-sen University. Their husbands (alleged fathers) were also included in the study. Three milliliters of peripheral blood were collected in EDTA-containing tubes. Amniotic fluid or chorionic villi were collected for medical purpose by professional operations at the hospital. Supplementary Table S1 shows the characteristics of the donors. All samples were anonymously collected after informed consent was obtained. All the experimental processes in this study strictly followed ethical research principles, and all methods were performed in accordance with the relevant guidelines and regulations. This study was approved by the Ethics Committee of Sun Yat-sen University (No. [2019]62).

For both the pregnant women and their husbands, blood stain papers were prepared. To obtain the DNA of the child, maternal plasma was isolated from peripheral blood by a two-step centrifugation method [24,25]. cfDNA from 1-milliliter plasma samples was extracted using the QIAamp Circulating Nucleic Acid Kit (Qiagen, Hilden, Germany) on a QIAvac 24 Plus vacuum manifold (Qiagen, Hilden, Germany) according to the manufacturer’s instructions and subsequently eluted to 20 microliters. Genomic DNA (gDNA) was extracted from the amniotic fluid using the QIAamp DNA Mini Kit (Qiagen, Hilden, Germany) while that from chorionic villus was extracted via the Chelex-100 method. The extracted DNA was quantified using the Qubit dsDNA HS Assay Kit (Thermo Fisher Scientific, San Francisco, CA, USA) with a Qubit 3.0 fluorometer (Thermo Fisher Scientific, San Francisco, CA, USA) according to the manufacturer’s instructions.

### 2.2. Library Preparation and Massively Parallel Sequencing

DNA libraries were prepared with 1 ng input DNA or two punched blood stained papers (1.0 mm^2^) using the ForenSeq DNA Signature Prep Kit (DNA Primer Mix A, DPMA; Verogen., San Diego, CA, USA) according to the manufacturer’s recommendations. Sequencing was performed on a MiSeq FGx system (Verogen., San Diego, CA, USA) using the MiSeq FGx Reagent Kit (Verogen., San Diego, CA, USA) following the manufacturer’s instructions.

### 2.3. Pedigree Confirmation by CE-Based STR Typing

Both parents and their fetus (amniotic fluid or chorionic villus) were genotyped using the Goldeneye 25A kit (Peoplespot, Beijing, China), which included 23 A-STR loci, according to the manufacturer’s instructions. Then, the PCR-products were separated on an AB 3500xL Genetic Analyzer (Thermo Fisher Scientific, San Francisco, CA, USA) and analyzed using the GeneMapper ID-X Version 1.5 (Thermo Fisher Scientific, San Francisco, CA, USA). Paternity index (PI) values were calculated according to the technical specification for the paternity test (GB/T 37223-2018) implemented in China [26]. Paternity inclusion was affirmed with a combined paternity index (CPI) value of greater than 10,000, whereas paternity exclusion was affirmed with a CPI of less than 0.0001.

### 2.4. Genotype Calling

**Figure 1:**
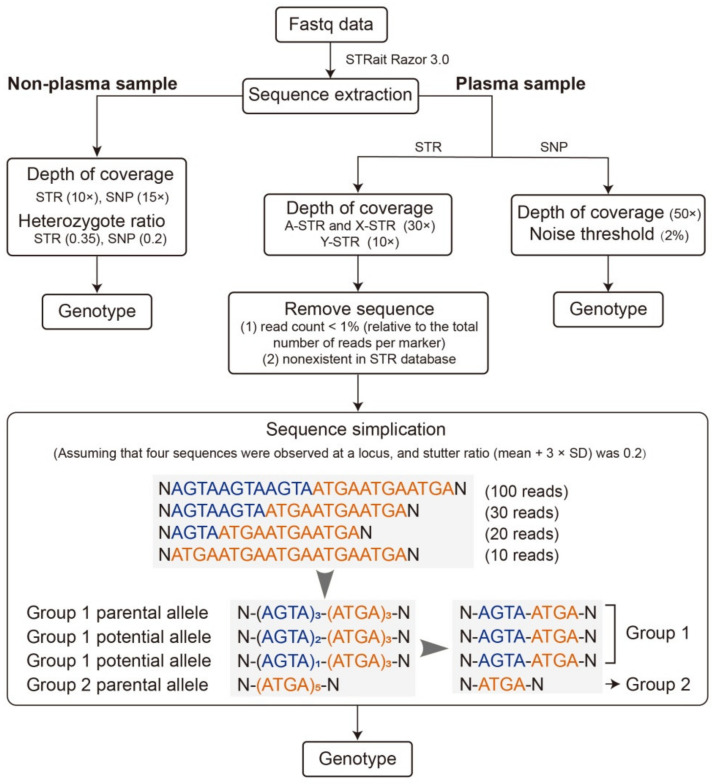
The workflow for genotype calling.

Genotype calling of SNP and STR markers was performed with STRait Razor 3.0 [27] and an in-house R script. The workflow for genotype calling is shown in Figure 1. For nonplasma samples, a threshold of the locus-specific depth of 15× and 10×, and a heterozygote ratio of 0.2 and 0.35 were applied for SNP and STR, respectively. Heterozygote ratio was calculated by dividing the lower coverage allele by the higher coverage allele at each locus.

For plasma samples, a minimum depth of 50× was required for SNP allele calling. We set 2% as the threshold to distinguish paternally derived alleles from background noise in cfDNA as studied before [20]. We also formulated a rule to define an expected paternally inherited allele (*e*PIA) and observed paternally inherited allele (*o*PIA). Specifically, when a fetus was identified to had an allele that the mother did not have at a locus, this allele was defined as an *e*PIA. For example, if the mother showed the homozygous genotype of AA and the fetus showed the heterozygous genotype of AB, allele B was an expected paternally derived allele, or to say *e*PIA. Then, if the plasma was also identified to have an allele that the mother did not have at that locus, this allele was defined as an *o*PIA. For the above example, if the number of reads of the minor SNP allele B in corresponding plasma exceeded the noise threshold, allele B was an observed paternally derived allele, or to say, *o*PIA, and the inferred genotype was supposed as AB.

For A-STR and X-STR allele calling in plasma samples, a minimum depth of 30× was required, while it was 10× for Y-STR. In STR analysis, *e*PIA and *o*PIA have the same definition as that in the analysis of SNPs, but the process of identifying alleles in plasma was more complicated. A novel strategy, inspired by our previous study about the characteristics of stutter variants in STR genotyping [23], was developed for the identification of STR alleles in plasma. Firstly, sequences with read counts of <1% were excluded for each locus, assuming that they were mainly PCR-derived noises or sequencing errors. Secondly, sequences that were not observed in the self-established STR sequence database, which consisted of 1527 alleles based on four Chinese populations, one Nigerian population [28], and four major American populations [29], were eliminated. All the alleles detected in nonplasma samples of both parents were added to the STR sequence database to avoid exclusion of novel alleles. Thirdly, a sequence simplification process for the remaining sequences was performed following Li et al.’s strategy [23]. According to this method, sequences with the same simplified architecture were categorized into one group and the sequence with the highest read count was considered as the parental allele of that group. For nonparental sequences within a specific group, sequences sharing the same simplified architecture as the parental allele were considered as potential paternal alleles if their read counts—(1) exceeded the mean plus three-fold of the stutter ratio standard deviation (SD) for alleles at the N − 1 and N + 1 positions; and (2) were higher than half the read counts of their corresponding parental alleles (e.g., for the sequence of N − 2 position, its corresponding parental allele is the sequence of N − 1 position) for alleles at other stutter positions. Finally, the genotype of the plasma was determined with a combination of the parental alleles and potential alleles at one locus for a specific sample. As a result, *o*PIAs were identified as the combination of the parental alleles and potential alleles minus maternal alleles.

### 2.5. Data Analysis

The dropout rate and drop-in rate for STRs and SNPs were obtained from a comparison between *o*PIAs and *e*PIAs. The fetal fraction in cfDNA was calculated using the formula:

(1) $$\frac{2\times n_{father}}{n_{father}+n_{mother}}$$

where $n_{\mathrm{father}}$ and $n_{\mathrm{mother}}$ represent the reads number of the *o*PIA and maternal allele for one SNP, respectively. The fetal fraction of a plasma was estimated by averaging the fractions of all loci. Detection rate of the *e*PIA for SNPs was computed as:

(2) $$\frac{n_{correct\;}}{n_{expected\;}}$$

where $n_{\mathrm{correct}\;}$ stands for the number of *o*PIAs consistent with paternal alleles of the reference fetus while $n_{\mathrm{expected}\;}$ stands for the number of *e*PIAs.

### 2.6. PI and CPI Calculation for NIPPT

To verify discrimination power of the system, the paternity of 16 unrelated subjects (the alleged fathers outside the studied family) were tested for each case, resulting in a total of 272 (16 alleged fathers × 17 families) negative tests. PI values were calculated using the R package relMix (Available online: http://cran.r-project.org/web/packages/relMix (accessed on 14 December 2020)), in which both mutations and the probabilities of dropout and/or drop-in were considered [30]. The CPI was calculated as the product of PIs and an LR threshold of 10,000, 1000, 100, and 10 was used as the threshold for confirmation of the parent–child relationship.

## 3. Results

### 3.1. Performance of the ForenSeq DNA Signature Prep Kit

**Figure 2:**
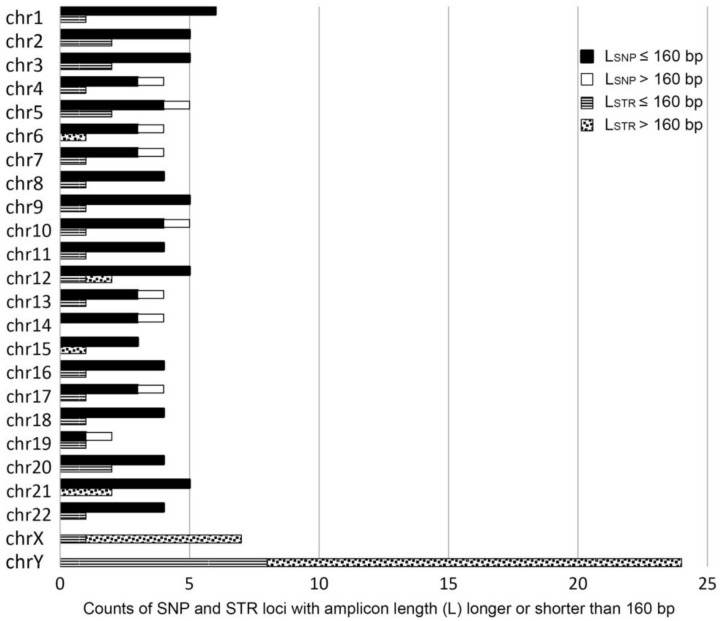
The density of single nucleotide polymorphisms (SNPs) and short tandem repeats (STRs) across different chromosomes and the number of SNP and STR loci with amplicon length (L) longer or shorter than 160 bp.

The ForenSeq DNA Signature Prep Kit (DPMA) allows targeted genotyping of 152 forensically relevant genetic markers in a single reaction, including 94 iiSNPs, 27 A-STRs, seven X-STRs, and 24 Y-STRs. As shown in Figure 2, these markers are diffusely distributed in different chromosomes, and the amplicon length of over 90% of SNP loci and nearly 80% of A-STR loci were shorter than 160 bp. However, for X-STR loci and Y-STR loci, approximately 86% and 66% of the loci exceeded 160 bp.

Prior to library generation, parent–offspring relationships were confirmed by STR genotyping with Goldeneye 25A system for all 17 families. Then, 68 samples from 17 families were sequenced with three sequencing runs. The average cluster density was 563 k/mm^2^ and the average percentage of the clusters passing filter, phasing, and prephasing were 97.95%, 0.17% and 0.11%, respectively. The average number of reads for each sample was 85,899.

### 3.2. PIA Identification from Plasma for NIPPT

For SNPs, a total of 313 *e*PIAs were expected in plasma across 17 families and the average number of *e*PIAs was 18 (range—10 to 22). The sequencing result of plasma showed that a total of 240 *o*PIAs were identified across 17 families with and the average number of *o*PIAs was 14 (range—1 to 21) (Table 1). That is, approximately 76.36% of paternal fetal SNP alleles could be detected on average. The fetal fraction in the plasma ranged from 0.32% to 50.3% (Supplementary Table S2). Full paternal fetal SNP alleles could be identified in cases with high fetal fractions; for example, in case 17 (12.57 gestational weeks; fetal fraction = 50.3%). In contrast, only one paternally derived fetal SNP allele was attained in case 14, which was at 7 gestational weeks with a fetal fraction of 0.32%. Among 240 *o*PIAs, 239 were consistent with profiles of the reference fetuses with one remaining false positive allele (fetal fraction = 4.69%) observed at rs2076848 in case 9. The overall error rate was 0.4%, similar to the error rate estimated by Christiansen et al. [10].

With respect to STRs, the average number of *e*PIAs for A-STRs, X-STRs, and Y-STRs was 17 (range—11 to 21), two (range—0 to 4), and three (range—0 to 24), respectively. The average number of *o*PIAs of A-STRs, X-STRs, and Y-STRs was eight (range—2 to 17), one (range—0 to 2), and 13 (range—11 to 15), respectively (Table 1). Twenty-three of 144 *o*PIAs at A-STRs were found to be false positives. Thereinto, 14, four, and five alleles resulted from N − 1 stutter, N + 1 stutter, and others (including stutters at other positions except for N − 1 and N + 1, microvariant allele, etc.), respectively (Supplementary Table S3). The drop-out rate and drop-in rate for A-STRs were 58% and 16%, respectively. For X-STR, 50% of *o*PIAs were found to be false positive and 75% of them resulted from N − 1 stutter. Y-STR profiles observed in the male fetuses of case 3 and case 21 totally matched those of biological fathers.

### 3.3. Influencing Factors on the Detection Rate

**Figure 3:**
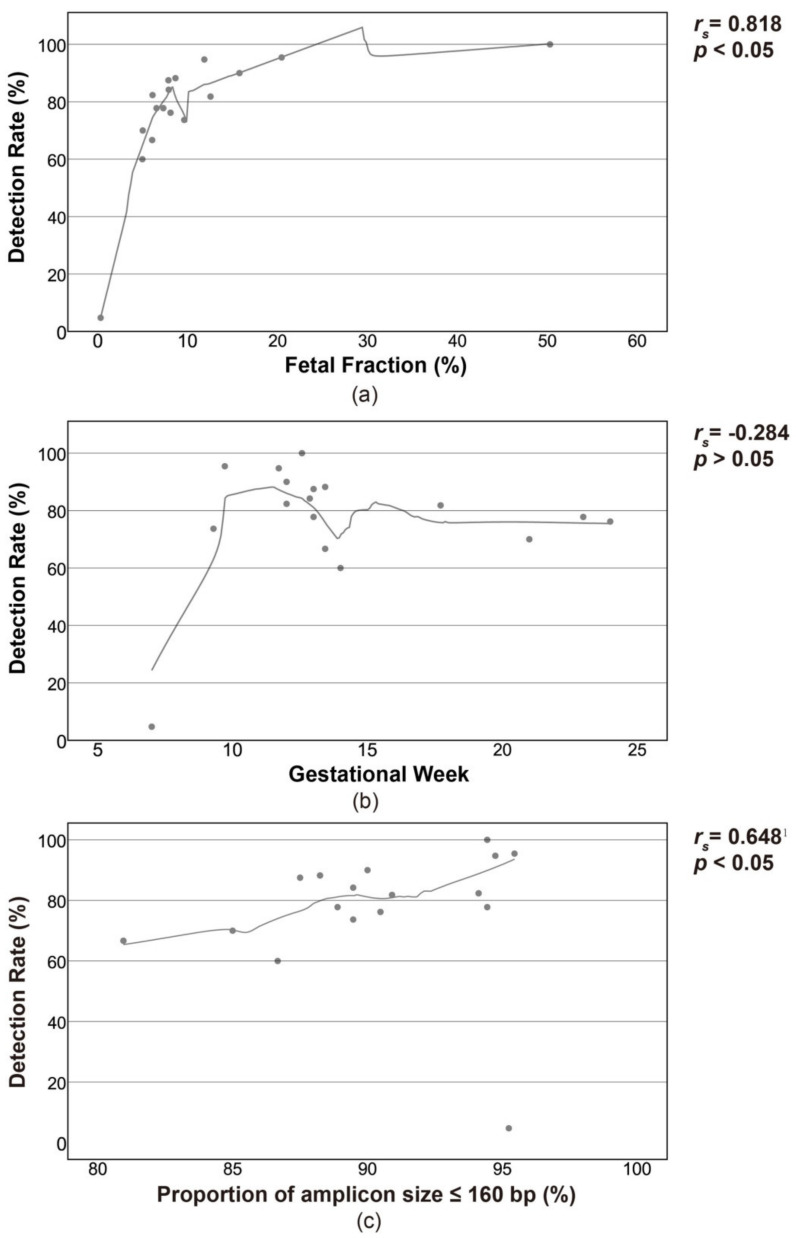
Correlation between detection rate for ePIAs and fetal fraction (**a**), gestational week (**b**) and amplicon size (**c**). ^1^ An outlier was excluded from statistical analysis.

For SNPs, the detection rate of *e*PIAs differed considerably, which ranged from 4.76% at case 14 to 100% at case 17. Also, the fetal fraction in the plasma varied greatly in a range between 0.32% and 50.3% (Supplementary Table S2). In order to explore the influencing factors of detection rate, dispersion plots were constructed using the value of the fetal fraction, length of the amplicon, and gestational week. As shown in Figure 3, the detection rate was correlated with fetal fraction and length of the amplicon, but not correlated with the gestational week.

### 3.4. CPIs with SNP and STR Typing for NIPPT

**Figure 4:**
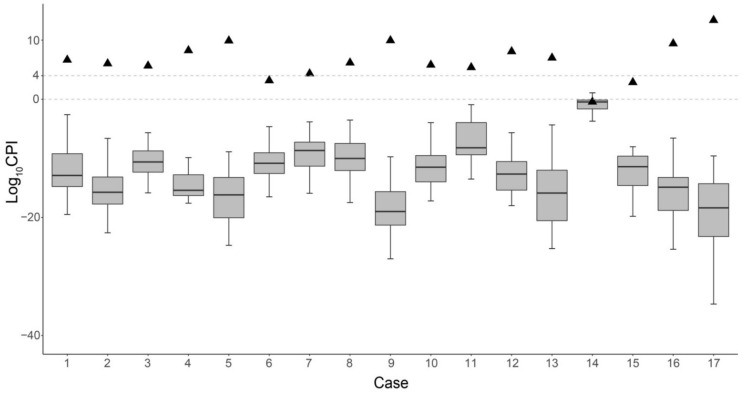
Log_10_CPI distribution in noninvasive prenatal paternity testing (NIPPT). Triangles represent the Log_10_CPI values obtained from 17 real trios. Boxplots show the distribution of Log_10_CPI values obtained from 272 unrelated trios.

The average Log_10_CPI value of the 17 families was 3.10 (range—0.28 to 6.27) when only 94 iiSNPs were applied and was 4.73 (range—−0.64 to 8.86) when only STRs were analyzed (Table 2). With an LR threshold of 10,000, 1000, 100, and 10 [31], a total of three, nine, 13, and 16 out of 17 family cases’ paternity could be determined using SNPs, respectively, and the values were six, 11, 14, and 14, respectively, when only A-STRs were used. X-STRs and Y-STRs were not considered for the PI calculation since little effective information could be obtained for cases presented in this study. As there is no significant LD in the Chinese population for these iiSNPs and A-STRs after Bonferroni correction [22,32], the PI values of iiSNPs and A-STRs were multiplied to calculate the CPI values. When combining the SNP and A-STR marker, 82.35% (14/17), 88.24% (15/17), 94.12% (16/17) and 94.12% (16/17) of real family cases could be accurately determined with an LR threshold for inclusion set as 10,000, 1000, 100 and 10, respectively (Table 3). The three cases with Log_10_CPI < 4 were case 6, case 14 and case 15, with the Log_10_CPI were 3.25, −0.36 and 2.94, respectively. As shown in Figure 4 and Table 2, the Log_10_CPI values obtained from 272 unrelated trios were all below four and separated significantly from the values of corresponding real trios except in case 14, which had the lowest fetal fraction and detection rate among 17 families.

## 4. Discussion and Conclusions

Paternity testing is often necessary in a wide range of situations to provide DNA evidence for the forensic community. Therein, antenatal paternity testing is of special use when investigating crimes related to rape or incest. Compared with invasive prenatal paternity testing by amniocentesis and chorionic villus sampling, noninvasive prenatal paternity testing based on cffDNA is less harmful and has stronger timeliness because testing could be conducted earlier, which reduces potential risks to both mother and fetus. However, most studies have focused on self-designed SNP typing systems with loci that have not been included in reference databases or commercial kits. Data communication and standard establishment were difficult, let alone the application of these panels in forensic practice. To take advantage of well-established STR markers, we investigated the utility of a standard forensic multiplex assay system, which enables genotyping of SNPs and STRs simultaneously, in NIPPT.

SNP typing results showed that, on average, 76.36% of paternal SNP alleles could be detected, reaching up to 14 *o*PIAs per case. Detection rates of *e*PIAs for SNPs varied among different cases but tended to increase with fetal fractions and proportions of short amplicons. Since cffDNA is mostly DNA fragments less than 160 bp, panels consisting of shorter amplicons will inevitably yield a higher success rate compare to that including a higher proportion of long amplicons. The strong correlation between detection rate and gestational week, which was reported in previous studies, was not observed in this work [10,20]. The most probable reason is that the pregnant women enrolled in the study were not from various gestational ages but concentrated at 12 to 14 gestational weeks, and the differences in detection rates were individual differences.

Although the existence of cfDNA in maternal plasma allows noninvasive prenatal paternity testing, fetal DNA generally comprises <20% of cfDNA in maternal plasma [13], which demands sensitive techniques and appropriate analytical methods to be detected. In the past years, SNP markers were mostly used in NIPPT due to their short amplicon sizes and absence of stutters [33]. However, thousands of markers are recommended to achieve higher accuracy, due to the limited polymorphisms of SNP markers [16]. Moreover, whether it is the widely-explored SNP marker or the newly-recruited microhaplotype marker, self-designed panels are unfavorable for data communication and utilization of public reference database, and usually lack evaluation. STR markers, by contrast, have multiple alleles and high information content and thus, are the gold standard marker for relationship analysis [34]. With the same number of markers, STRs are superior to SNPs in conventional parentage testing. However, the length of amplicons in CE typing and the presence of stutters both limited the application of STR markers in NIPPT [11,12]. STR markers are the main markers for the forensic community, both now and in the future. If the advancement of well-established STR markers could be taken, the application of NIPPT could be broadened. In the present study, ForenSeq DNA Signature Prep Kit, which targeting well-established STRs and SNPs, and with shorter amplicons than the common CE typing method, was applied for NIPPT for the purpose of increasing the STR genotyping success rate. Besides, investigation of paternally derived fetal STR alleles was performed with a novel strategy, which may bring improvement for mixture deconvolution under stutter interference [23]. However, the result demonstrated that there still was a risk of misidentifying sequencing error or stutter artifacts as paternal alleles due to the complexity of STR variations and the shortage of PCR-based methods. As the molecular architecture of STRs contributes to the generation of stutters [35], including more A-STRs to improve discriminating power may become a solution. Furthermore, a bioinformatics pipeline based on unique molecular identifier (UMI) technology was developed recently, which allows PCR or sequencing errors to be identified efficiently and, subsequently, increase the accuracy of allele interpretation during MPS-based STR genotyping [36]. Therefore, recruitment of this method in NIPPT with well-established STR markers could be conducted in the next step.

The result also showed that a single type of marker in the ForenSeq DNA Signature Prep Kit may not be sufficient for NIPPT, and a combination of multiple types of markers (SNPs and STRs) will improve the testing efficiency. For the three cases with paternity that could not be determined with the LR threshold of 10,000, the stage of pregnancy should be the main reason. In fact, they are amongst the earliest gestational weeks within this study. The earlier the gestational age, the higher probability of low fetal fraction in the plasma, which would further determine a lower *o*PIA detection rate and, subsequently, low CPI. Therefore, when using this panel to do NIPPT for women at early gestational weeks, false negatives may occur, and it should be more cautious to exclude paternity. Even so, this study still indicated that the ForenSeq DNA Signature Prep Kit could solve a high proportion of NIPPT cases.

However, the application of NIPPT should be very cautious. From a technical aspect, either false negative or false positive PIAs might occur, which will further affect the testing result and make it inconclusive. In this condition, additional tests must be done before determining paternity inclusion or exclusion. Furthermore, ethical and social issues should always be considered [37,38,39,40]. One of the major aspects to consider is the protection of under-age children who are not able to express their intention and are vulnerable in the face of setbacks [37]. For noninvasive paternity testing which involves embryos, both rights of the embryos and the parents should be protected. Therefore, the testing should be conducted even more cautiously, especially in countries where termination of pregnancy is allowed for nonmedical reasons. The participants’ ability to deal with the outcome of NIPPT should be considered, and they should always be warned of the potential implications of such testing. Overall, NIPPT should be conducted within the context that highly sensitive and accurate NIPPT methods are developed and ethical aspects are fully considered. To our knowledge, this is the first study exploring the utilization of the ForenSeq DNA Signature Prep Kit in NIPPT. It revealed that NIPPT was possible with a combination of well-established SNPs and STRs. The results of this study may facilitate the standardization of NIPPT in forensic practice. However, more efforts are needed on the selection of effective markers and development of interpretation strategies, to aid in the application of NIPPT in forensic applications.

## Figures and Tables

**Table 1:** Number of expected paternally inherited allele *(e*PIAs) and observed paternally inherited allele (*o*PIAs) identified in maternal plasma samples.

Case	SNPs		A-STRs		X-STRs		Y-STRs	
	*e*PIAs	*o*PIAs	*e*PIAs	*o*PIAs	*e*PIAs	*o*PIAs	*e*PIAs	*o*PIAs
1	18	14	21	7 + 1 ^1^	2	1 ^1^	0	0
2	21	16	21	9 + 2 ^1^	0	0	27	11
3	21	14	20	7 + 2 ^1^	3	0	0	0
4	17	15	19	10	4	1 + 1 ^1^	0	0
5	19	16	18	9	1	1	0	0
6	18	14	19	3 + 3 ^1^	4	0	0	0
7	10	9	16	7 + 3 ^1^	3	1^1^	0	0
8	17	14	14	4	2	1^1^	0	0
9	19	18 + 1 ^1^	16	8 + 2 ^1^	4	1	0	0
10	16	14	14	5 + 2 ^1^	4	0	0	0
11	15	9	16	5 + 1 ^1^	3	2 ^1^	0	0
12	20	14	11	7	3	1	0	0
13	22	18	17	8 + 2 ^1^	3	1 + 2 ^1^	0	0
14	21	1	15	1 + 1 ^1^	2	0	0	0
15	19	14	17	4 + 3 ^1^	3	1	0	0
16	22	21	13	10 + 1 ^1^	0	0	27	15
17	18	18	19	17	2	2	0	0

^1^ False positive *o*PIA, which is inconsistent with the profile of the reference fetus.

**Table 2:** Log_10_CPI values for 17 families.

Case	Sum of *o*PIAs for SNPs and A-STRs	Log_10_CPI Based on SNP Typing	Log_10_CPI Based on A-STR Typing	Log_10_CPI Based on SNP and A-STR Typing
1	22	2.91	3.82	6.73
2	27	2.65	3.47	6.12
3	23	1.78	3.96	5.74
4	25	3.86	4.48	8.34
5	25	3.74	6.25	9.99
6	20	3.08	0.17	3.25
7	19	1.47	2.95	4.42
8	18	3.92	2.34	6.26
9	29	6.27	3.76	10.03
10	21	3.50	2.38	5.88
11	15	1.79	3.68	5.47
12	21	3.39	4.78	8.17
13	28	2.63	4.47	7.10
14	3	0.28	−0.64	−0.36
15	21	2.24	0.70	2.94
16	32	4.42	5.08	9.50
17	35	4.60	8.86	13.46

**Table 3:** Combined paternity index (CPI) distribution for real trios and unrelated trios with the combination of SNPs and autosomal STRs (A-STRs).

LR Threshold	Real Parentage (*n* = 17)	Unrelated (*n* = 272)
>10	94.12%	0.36%
>100	94.12%	0
>1000	88.24%	0
>10,000	82.35%	0

## Data Availability

The data presented in this study are available on request from the corresponding author. The data are not publicly available due to the restriction of privacy.

## Supplementary Materials

The following are available online at https://www.mdpi.com/2073-4425/12/3/454/s1, Table S1: Characteristics of the donors, Table S2: Statistical parameters of cases in the test, Table S3: Characteristics of false positive *o*PIAs.
