# Supplementary material for: Noninvasive Prenatal Paternity Testing with a Combination of Well-Established SNP and STR Markers Using Massively Parallel Sequencing
Source: Genes (Basel). 2021 Mar 22;12(3):454. doi: 10.3390/genes12030454 (PMC8004970; doi:10.3390/genes12030454)
Supplement: Supplementary file 1 [file genes-12-00454-s001.zip › Table S2.docx]

**Table S2.** Statistical parameters of cases in the test.

| Case | Gestational Week | Proportion of amplicon size ≤ 160 bp (%) | Fetal Fraction (%) | Detection Rate of *e*PIAs for SNPs (%) |
| --- | --- | --- | --- | --- |
| 1 | 23 | 88.89 | 6.53 | 77.78 |
| 2 | 24 | 90.48 | 8.09 | 76.19 |
| 3 | 13.43 | 80.95 | 6.05 | 66.67 |
| 4 | 13.43 | 88.24 | 8.64 | 88.24 |
| 5 | 12.86 | 89.47 | 7.89 | 84.21 |
| 6 | 13 | 94.44 | 7.29 | 77.78 |
| 7 | 12 | 90.00 | 15.76 | 90.00 |
| 8 | 12 | 94.12 | 6.09 | 82.35 |
| 9 | 11.71 | 94.74 | 11.85 | 94.73 |
| 10 | 13 | 87.50 | 7.86 | 87.50 |
| 11 | 14 | 86.67 | 4.96 | 60.00 |
| 12 | 21 | 85.00 | 5.00 | 70.00 |
| 13 | 17.71 | 90.91 | 12.54 | 81.82 |
| 14 | 7 | 95.24 | 0.32 | 4.76 |
| 15 | 9.29 | 89.47 | 9.61 | 73.68 |
| 16 | 9.71 | 95.45 | 20.45 | 95.45 |
| 17 | 12.57 | 94.44 | 50.30 | 100.00 |

* Conversion was made from days to week.
