# Supplementary material for: Noninvasive Prenatal Paternity Testing with a Combination of Well-Established SNP and STR Markers Using Massively Parallel Sequencing
Source: Genes (Basel). 2021 Mar 22;12(3):454. doi: 10.3390/genes12030454 (PMC8004970; doi:10.3390/genes12030454)
Supplement: Supplementary file 1 [file genes-12-00454-s001.zip › Table S3.docx]

**Table S3.** Characteristics of false positive *o*PIAs.

| Case | SNPs | A-STRs | | | X-STRs | | | Y-STRs | | |
| --- | --- | --- | --- | --- | --- | --- | --- | --- | --- | --- |
|  |  | N − 1 stutter | N + 1 stutter | Others^1^ | N − 1 stutter | N + 1 stutter | Others ^1^ | N − 1 stutter | N + 1 stutter | Others ^1^ |
| 1 | 0 | 1 | 0 | 0 | 1 | 0 | 0 | - | - | - |
| 2 | 0 | 1 | 1 | 0 | - | - | - | 0 | 0 | 0 |
| 3 | 0 | 1 | 1 | 0 | 0 | 0 | 0 | - | - | - |
| 4 | 0 | 0 | 0 | 0 | 1 | 0 | 0 | - | - | - |
| 5 | 0 | 0 | 0 | 0 | 0 | 0 | 0 | - | - | - |
| 6 | 0 | 2 | 1 | 0 | 0 | 0 | 0 | - | - | - |
| 7 | 0 | 0 | 1 | 2 | 1 | 0 | 0 | - | - | - |
| 8 | 0 | 0 | 0 | 0 | 1 | 0 | 0 | - | - | - |
| 9 | 1 | 2 | 0 | 0 | 0 | 0 | 0 | - | - | - |
| 10 | 0 | 2 | 0 | 0 | 0 | 0 | 0 | - | - | - |
| 11 | 0 | 0 | 0 | 1 | 1 | 0 | 1 | - | - | - |
| 12 | 0 | 0 | 0 | 0 | 0 | 0 | 0 | - | - | - |
| 13 | 0 | 0 | 0 | 2 | 1 | 0 | 1 | - | - | - |
| 14 | 0 | 1 | 0 | 0 | 0 | 0 | 0 | - | - | - |
| 15 | 0 | 3 | 0 | 0 | 0 | 0 | 0 | - | - | - |
| 16 | 0 | 1 | 0 | 0 | - | - | - | 0 | 0 | 0 |
| 17 | 0 | 0 | 0 | 0 | 0 | 0 | 0 | - | - | - |

^1^ Represents variants including stutters at other positions except N − 1 and N + 1, microvariant allele, etc.
