# Supplementary material for: Noninvasive Prenatal Paternity Testing with a Combination of Well-Established SNP and STR Markers Using Massively Parallel Sequencing
Source: Genes (Basel). 2021 Mar 22;12(3):454. doi: 10.3390/genes12030454 (PMC8004970; doi:10.3390/genes12030454)
Supplement: Supplementary file 1 [file genes-12-00454-s001.zip › Table S1.docx]

**Table S1.** Characteristics of the donors.

| Case | Sample ID ^1^ | Origin | Gestation Week | Sex | Clinical state |
| --- | --- | --- | --- | --- | --- |
| 1 | WC1 | Maternal plasma | 23 |  |  |
|  | WC1F | Father’s blood spot |  | Male | No special |
|  | WC1M | Mother’s blood spot |  | Female | No special |
|  | WC1Z | Amniotic fluid |  | Female | No special |
| 2 | WC3 | Maternal plasma | 24 |  |  |
|  | WC3F | Father’s blood spot |  | Male | No special |
|  | WC3M | Mother’s blood spot |  | Female | No special |
|  | WC3Z | Amniotic fluid |  | Male | No special |
| 3 | WC4 | Maternal plasma | 13 + 3 |  |  |
|  | WC4F | Father’s blood spot |  | Male | No special |
|  | WC4M | Mother’s blood spot |  | Female | No special |
|  | WC4Z | Amniotic fluid |  | Female | No special |
| 4 | WC5 | Maternal plasma | 13 + 3 |  |  |
|  | WC5F | Father’s blood spot |  | Male | No special |
|  | WC5M | Mother’s blood spot |  | Female | No special |
|  | WC5Z | Amniotic fluid |  | Female | No special |
| 5 | WC8 | Maternal plasma | 12 + 6 |  |  |
|  | WC8F | Father’s blood spot |  | Male | No special |
|  | WC8M | Mother’s blood spot |  | Female | No special |
|  | WC8Z | Amniotic fluid |  | Female | No special |
| 6 | WC9 | Maternal plasma | 13 |  |  |
|  | WC9F | Father’s blood spot |  | Male | No special |
|  | WC9M | Mother’s blood spot |  | Female | No special |
|  | WC9Z | Amniotic fluid |  | Female | No special |
| 7 | WC10 | Maternal plasma | 12 |  |  |
|  | WC10F | Father’s blood spot |  | Male | No special |
|  | WC10M | Mother’s blood spot |  | Female | No special |
|  | WC10Z | Amniotic fluid |  | Female | No special |
| 8 | WC11 | Maternal plasma | 12 |  |  |
|  | WC11F | Father’s blood spot |  | Male | No special |
|  | WC11M | Mother’s blood spot |  | Female | No special |
|  | WC11Z | Amniotic fluid |  | Female | No special |
| 9 | WC12 | Maternal plasma | 11 + 5 |  |  |
|  | WC12F | Father’s blood spot |  | Male | No special |
|  | WC12M | Mother’s blood spot |  | Female | No special |
|  | WC12Z | Amniotic fluid |  | Female | No special |
| 10 | WC14 | Maternal plasma | 13 |  |  |
|  | WC14F | Father’s blood spot |  | Male | No special |
|  | WC14M | Mother’s blood spot |  | Female | No special |
|  | WC14Z | Amniotic fluid |  | Female | No special |
| 11 | WC15 | Maternal plasma | 14 |  |  |
|  | WC15F | Father’s blood spot |  | Male | No special |
|  | WC15M | Mother’s blood spot |  | Female | No special |
|  | WC15Z | Amniotic fluid |  | Female | No special |
| 12 | WC16 | Maternal plasma | 21 |  |  |
|  | WC16F | Father’s blood spot |  | Male | No special |
|  | WC16M | Mother’s blood spot |  | Female | No special |
|  | WC16Z | Amniotic fluid |  | Female | No special |
| 13 | WC17 | Maternal plasma | 17 + 5 |  |  |
|  | WC17F | Father’s blood spot |  | Male | No special |
|  | WC17M | Mother’s blood spot |  | Female | No special |
|  | WC17Z | Amniotic fluid |  | Female | No special |
| 14 | WC19 | Maternal plasma | 7 |  |  |
|  | WC19F | Father’s blood spot |  | Male | Fertility treatment |
|  | WC19M | Mother’s blood spot |  | Female | Fertility treatment |
|  | WC19Z | Chorionic villus |  | Female | No special |
| 15 | WC20 | Maternal plasma | 9 + 2 |  |  |
|  | WC20F | Father’s blood spot |  | Male | Fertility treatment |
|  | WC20M | Mother’s blood spot |  | Female | Fertility treatment |
|  | WC20Z | Chorionic villus |  | Female | No special |
| 16 | WC21 | Maternal plasma | 9 + 5 |  |  |
|  | WC21F | Father’s blood spot |  | Male | Fertility treatment |
|  | WC21M | Mother’s blood spot |  | Female | Fertility treatment |
|  | WC21Z | Chorionic villus |  | Male | No special |
| 17 | WC22 | Maternal plasma | 12 + 4 |  |  |
|  | WC22F | Father’s blood spot |  | Male | Fertility treatment |
|  | WC22M | Mother’s blood spot |  | Female | Fertility treatment |
|  | WC22Z | Chorionic villus |  | Female | No special |

^1^ The characters F, M and Z represent father, mother, and their child, respectively.
